# Abnormal Lipid levels as a risk factor of eclampsia, study conducted in tertiary care Hospitals of Khyber Pakhtunkhwa Province - Pakistan

**DOI:** 10.12669/pjms.296.3863

**Published:** 2013

**Authors:** Rubina Nazli, Muhammad Akmal Khan, Tasleem Akhtar, Ghosia Lutfullah, Nabila Sher Mohammad, Jawad Ahmad, Jamila Haider, Hina Aslam

**Affiliations:** 1 Rubina Nazli, MBBS, DGO, PhD, Institute of Basic Medical Sciences, Khyber Medical University, KPK Peshawar, Pakistan.; 2 Muhammad Akmal Khan, MBBS, FCPS, MRCP, Hayatabad Medical Complex, Peshawar, Pakistan.; 3 Tasleem Akhtar, M.Phil, PhD, PMRC Research Centre Khyber Medical College Peshawar, Pakistan.; 4 Ghosia Lutfullah, M.Phil, PhD, Centre of Biotechnology & Microbiology, University of Peshawar, KPK Peshawar, Pakistan.; 5 Nabila Sher Mohammad, MBBS, Khyber Girls Medical College, KPK Peshawar, Pakistan.; 6 Jawad Ahmad, MBBS, PhD, Institute of Basic Medical Sciences, Khyber Medical University, KPK Peshawar, Pakistan.; 7 Jamila Haider, M.Phil scholar, Centre of Biotechnology & Microbiology, University of Peshawar, KPK Peshawar, Pakistan.; 8 Hina Aslam, MBBS, Khyber Girls Medical College, KPK Peshawar, Pakistan.

**Keywords:** Eclampsia, Hypertension, Lipoprotein, Lipoprotein ratio, Khyber Pakhtunkhwa Province

## Abstract

***Objective:*** To evaluate abnormal lipid metabolism as a risk factor of eclampsia in pregnant women.

***Methods:*** This cross sectional study was conducted in three tertiary care hospitals of Peshawar. Serum total cholesterol (TC), high density lipoprotein cholesterol (HDL-C), low density lipoprotein cholesterol (LDL-C), very low density lipoprotein cholesterol (VLDL-C), triglyceride (TG), apolipoprotein A1 (APO-A1), APO-B100, lipoprotein-a (Lpa) were measured in 110 women with eclampsia and compared with 90 healthy pregnant women. Mean lipid levels in cases and controls were compared using student’s t test”.

***Results:*** Mean systolic/diastolic blood pressure, TC, TG, VLDL-C and Lpa levels were significantly higher (p<0.001) in patients compared to control women. Similarly TC: HDL-C, LDL-C: HDL-C and TG: HDL-C ratio in the patients group were significantly higher (p<0.001) and HDL-C: VLDL-C ratio was significantly lower (p<0.001) in the patients as compared to control group. Undesirable cholesterol were noted in 35.8% patients, HDL-C in 50.5%, borderline high concentration of LDL-C in 23.6%, high triglycerides levels in 73.2%, undesirable cholesterol ratio in 52.3% and undesirable LDL-C ratio were noted in 82.1% patients of eclampsia.

***Conclusion:*** Serum lipids were found significantly higher thus early assessment may be helpful in prevention of complications in the eclampsia patients.

## INTRODUCTION

An acute and life-threatening complication of pregnancy is eclampsia characterized by the start of tonic-clonic seizures in a patient associated with proteinuria and is a most important cause of maternal and perinatal mortality. In developing countries^1^, the prevalence of eclampsia varies widely, from 1 in 100 to 1 in 1700. The prevalence of eclampsia, reported from various parts of Pakistan^2^ is ranging from 1.6% to 3.1%. In Pakistan^3^; maternal death from eclampsia is reported as 9 to 16.9%.

The association of variations of serum lipid profile in essential hypertension is well documented. Disorders of the lipoprotein metabolism are an important cause of endothelial dysfunction that results in hypertension and proteinuria, clinical hallmarks of pregnancy-induced hypertension.^4^ Increased triglyceride levels found in pregnancy-induced hypertension is possible to be deposited in predisposed vessels, such as the uterine spiral arteries and contributes to the endothelial dysfunction, both directly and indirectly through generation of small, dense LDL-C.^5^

The purpose of this study was to determine and compare the serum lipid levels among women with eclampsia and women having normal pregnancy without hypertension in Khyber Pakhtunkhwa Province, Pakistan.

## METHODS

This cross sectional study was undertaken on 200 pregnant women at gestational age > 20 weeks. 110 pregnant women with eclampsia admitted in the Gynea and Obstrectics units of three teaching hospitals of Peshawar, Khyber Pakhtunkhwa Province-Pakistan were randomly selected. From the same health facilities, 90 healthy pregnant women without a history of hypertension were randomly selected as control group for comparison. After explaining aims and objectives, informed consent was obtained from each subject for participation in this study. Ethical approval for the study was obtained from the Institutional Ethical Research Board (IERD) at Post Graduate Medical Institute, Hayatabad Medical complex, Peshawar.

Eclampsia was defined as the occurrence of hypertension at > 20 weeks of gestation with proteinuria, edema, tonic-clonic convulsions/coma and with systolic blood pressure > 140 mm Hg and diastolic blood pressure > 90 mm Hg on repeated readings.

Patient’s information like maternal age, parity and gestation age at screening was recorded. Blood pressure, height and weight of the participants were measured at the time of enrolment, by using standard methods.

Venous blood samples were obtained from both patients and controls in non fasting state. Biochemical analysis of lipoprotein included total cholesterol, high density lipoprotein cholesterol, low density lipoprotein cholesterol, very low density lipoprotein cholesterol, triglycerides, APO-A1, APO-B100 and lipoprotein-a level.

The data was processed on computer software package SPSS version 11. The numerical data was presented as mean + SEM. The Student’s t test was used to evaluate mean differences in maternal serum lipid concentrations between patients and control subjects. Significance among the means of groups was expressed in term of ‘P’ value. 95% Confidence Interval (P < 0.05) was considered as significant.

## RESULTS

The demographic and reproductive characteristics of the patient and control groups are compared in Table-I. The age of patients and control group was comparable. Mean parity was significantly (p<0.01) lower and mean systolic/diastolic blood pressure was significantly higher (p<0.001) in women of eclampsia group as compared to healthy pregnant women. Difference between gestation age and the Body Mass Index (BMI) was non-significant among the groups.

Table-II compares mean (+ SEM) lipoprotein concentration among the study groups. Highly significant (p < 0.001) differences were noted in most of maternal serum lipids in the patients group except changes in LDL-cholesterol, mean levels of total cholesterol and APO-B100 when compared with the control group. Eclampsia patients had 7.1%, 2.9%, 58.5%, 59.1, and 40% higher mean concentrations of total cholesterol, LDL cholesterol, VLDL cholesterol, triglycerides and Lpa respectively, as compared to control subjects.

**Table-I:** Socio-demographic and reproductive characteristics of the study groups

*Variables*	*Patients* *Mean * *+* * SEM*	*Control* *Mean * *+* * SEM*	*P value*
Age (years)	29.54+ 0.68	30.04+0.66	NS
Gestation age (weeks)	31.83 + 0.45	29.72 + 0.55	NS
Parity (n)	2.78 + 0.35	4.04 + 0.27	0.01
Systolic BP (mm Hg)	155.58 +1.97	116 + 0.91	0.001
Diastolic BP (mm Hg)	103.66 + 1.53	75.28 + 0.91	0.001
BMI (kg/m^2^)	29.27 + 1.12	28.94 + 0.82	NS

**Table-II:** Lipoprotein concentrations among the study groups of pregnant women

*Lipoprotein (mg/dl)*	*Patients* *Mean* *+* * SEM*	*Control* *Mean* *+* * SEM*	*% change* *from control*	*P value*
Total Cholesterol	216.63+5.17	202.23+4.6	7.1↑	NS
HDL-Cholesterol	40.83 +0.92	52.20 +1.14	21.7 ↓	0.001
LDL-Cholesterol	110.54 +4.79	107.42 +4.07	2.90 ↑	NS
VLDL-Cholesterol	66.93 +2.84	42.22 +1.46	58.5↑	0.001
Triglyceride	337.71 +14..34	212.30 +7.28	59.1↑	0.001
APO-A1	144.43+4.50	190.14 +5.31	24.0↓	0.001
APO-B100	120.06+4.32	128.33 +5.50	6.4↓	NS
Lpa	81.62+5.61	58.3 +4.94	40.0↑	0.001

**Table-III:** Comparison of lipoprotein ratio among the patient and control groups

*Lipoprotein ratio*	*Patients* *Mean* *+* * SEM*	*Control* *Mean* *+* * SEM*	*% change* *from control*	*P value*
TC:HDL-C	5.49 +0.15	3.87+0.11	41.9↑	0.001
LDL-C:HDL-C	2.77 +0.12	2.05+0.09	79.0↑	0.001
TG:HDL-C	8.70+0.45	4.35+0.19	100↑	0.001
HDL-C: VLDL-C	0.71+0.03	1.36+0.06	47.8↓	0.001
LDL: APO-B100	1.02+0.06	0.81+0.05	25.9↑	0.01
HDL:APO-A1	0.31+0.01	0.28+0.012	10.7↑	NS
Apo B100: Apo-A1	0.83+ 0.96	0.67+1.03	23.9↑	NS

**Table-IV:** Comparison in different categories of lipoprotein concentrations among the study groups

*Lipid Concentration (mg/dl)*		*Patients %*	*Control %*
*Cholesterol*	*Category*		
< 200	Desirable	41.3	53.8
200-239	High	22.9	23.1
> 240	Undesirable	35.8	23.1
*HDL-C*			
> 60	Desirable	4.7	18.9
40-60	Acceptable	44.9	67.8
< 40	Undesirable	50.5	13.3
*LDL-C*			
< 100	Optimal	41.5	51.1
100-129	Near optimal	21.7	22.2
130-159	Borderline high	23.6	11.1
160-189	High	6.6	7.8
> 190	Very high	6.6	7.8
*VLDL-C*			
< 129	Optimal	95.4	100
130-159	Borderline high	3.7	-
> 160	High	0.9	-
*Triglyceride*			
0-149	Normal	4.6	14.6
150-199	Borderline high	6.5	34.8
200-499	High	73.2	49.4
> 500	Very high	15.7	1.1
*TC:HDL-C ratio*			
< 5.1	Desirable	47.7	85.6
> 5.1	Undesirable	52.3	14.4
*LDL-C:HDL-C ratio*			
< 1.21	Desirable	9.4	18.9
1.21-1.6	Acceptable	8.5	21.1
> 1.6	Undesirable	82.1	60.0
*HDL-C: VLDL-C ratio*			
< 3.3	Desirable	100	98.9
> 3.3	Undesirable	-	1.1

Comparison of lipoprotein ratio among the groups is given in Table-III. Total cholesterol: HDL ratios, LDL-C: HDL-C and TG: HDL-C ratios were higher (41.9%, 79.0% & 100% respectively) among the patients group and were highly significant (p< 0.001) as compared to normotensive pregnant women. In the patients group LDL: APO-B100 was raised to a significant level of p < 0.01, while the difference between the two groups was found non significant for HDL: APO-A1 ratio (p > 0.05). HDL-C: VLDL-C ratios were decreased by 47.8% in the patients and were highly significant as compared with control subjects (p < 0.001). APO-B 100: APO-A1 ration was found non significant among the groups.

Table-IV represents comparison between different categories of lipid concentrations as desirable, acceptable and undesirable among the study groups. Highest concentration of cholesterol greater than 240 mg/dl (undesirable) was noted in 35.8% patients of eclampsia group, while in more than 50% of the patients in the eclampsia group had HDL cholesterol which is considered to be undesirable. Borderline high concentration of LDL (23.6%), high concentrations of triglycerides (73.2%), undesirable cholesterol to HDL ratio > 5.2 in 52.3%, undesirable LDL to HDL ratio > 1.6 were noted in 82.1% patients of eclampsia.

## DISCUSSION

Worldwide diverse studies have reported^6^ elevated lipid levels in pregnancy induced hypertension patients. Some earlier studies reported that the striking changes in the lipid profile in normal pregnancy is serum hypertriglyceridemia, which may be as high as two to three folds in the third trimester over the levels in non pregnant women.^7^ In our study also this observation holds true and the rise in serum triglycerides was statistically significant (P<0.001) in eclampsia patients when compared to women with normal pregnancy.

In the present study, cholesterol concentration increased in the patients of eclampsia to a non significant level of p=0.05. These results are consistent with the studies conducted in Pakistan^8^^,^^9^ and other populations.^10^ It was reported in the Pakistani^8^^-^^9^ and Peruvian populations^11^ that patients with pregnancy induced hypertension had higher mean triglyceride and lower mean serum HDL-C concentrations than the control group. In our study, the mean values of HDL-C were about 21.7% lower and triglycerides 59.1% higher respectively in the patients over the pregnant women with normal pregnancy. Statistically the variation were highly significant (P<0.001). The change in LDL-C cholesterol was non significant in the two groups of patient and control that is contrary to a report from Pakistani population.^9^

In the present study, APO-B100 concentration in the patients was lower when compared to control group. A significant difference in APO-A1 levels was seen in eclampsia group in contrast to that of control group. In our view, this can be originated from polymorphism of APO-A1 of HDL-C and/or functional disorder of HDL-C. Similar results are reported by Bayhan G et al from Turkey.^12^

In women with eclampsia we noted significantly higher Lpa concentrations in contrast to control group. Our results are in accordance to other studies^13^ which shows elevated Lpa level in women with pregnancy induced hypertension but dissimilar to a study^14^ presenting lower Lpa levels in women with pregnancy induced hypertension in contrast to healthy pregnant controls. Lipoprotein (a) can work as acute phase reactant in the presence of endothelial dysfunction or inflammation.

According to different studies, LDL-C: HDL-C ratio increased significantly in eclamptic women as compared to normal pregnant women.^15^ On the other hand TG: HDL-C increased significantly in eclampsia. Trend was slightly different in TC: HDL-C which decreased during pregnancy but increased significantly in eclampsia.^16^ Though the relevance of these ratios in pregnancy and eclampsia is yet to be established, the significance of altered TC: HDL-C, TG: HDL-C and HDL-C: VLDL-C ratios cannot be overlooked as they indicate additional risks in eclampsia. Although it has recently been suggested that in the progression of CVD apolipoproteins may be more informative risk markers than lipoproteins like LDL and HDL^17^ particularly, the ratio between apolipoprotein B and apolipoprotein A-I (apoB/apoA-I).^18^^,^^19^ The significance of LDL: Apo B-100 and HDL: Apo-A1 and their possible relationship with eclampsia is yet not clear.

These findings are in accordance with outcome from the few existing prospective cohort studies^20^, and many case-control studies^21^ of maternal fasting or non fasting plasma lipid and lipoprotein concentrations in pregnancy induced hypertension and normotensive pregnancies.

## CONCLUSION

In this study abnormal lipid levels have been noted in the patients of eclampsia. It is therefore, essential that, blood lipid concentrations be estimated in pregnant women during antenatal care since it could be useful in the early prevention of obstetric complications of eclampsia.

***Limitation of study: ***The study was conducted on admitted patients of eclampsia. Owing to the patient condition serum lipoprotein and apo-lipoprotein were measured in non fasting state. More accurate results could be drawn if these investigations could be done in fasting state.

***Recommendations: ***The present study demonstrated that the blood levels for some serum lipid/lipoprotein were significantly changed in women with eclampsia. Since these high cardiac risk factors could result in severe problems including atherosclerosis and CHD. As scuh consideration should be given to monitoring such women for these cardiac risk factors both during pregnancy, as well as later in life.
